# Homeostasis imbalance process ontology: a study on COVID-19 infectious processes

**DOI:** 10.1186/s12911-024-02516-0

**Published:** 2024-05-22

**Authors:** Yuki Yamagata, Tatsuya Kushida, Shuichi Onami, Hiroshi Masuya

**Affiliations:** 1 Life Science Data Sharing Unit, Infrastructure Research and Development Division, RIKEN Information R&D and Strategy Headquarters, 2-2-3 Minatojima-minamimachi, Chuo-ku, Kobe, Hyogo 650-0047 Japan; 2 Laboratory for Developmental Dynamics, RIKEN Center for Biosystems Dynamics Research, 2-2-3 Minatojima-minamimachi, Chuo-ku, Kobe, Hyogo 650-0047 Japan; 3 Integrated Bioresource Information Division, RIKEN BioResource Research Center, 3-1-1 Koyadai, Tsukuba-shi, Ibaraki, 305-0074 Japan

**Keywords:** Ontology, COVID-19, Homeostasis imbalance, Infectious process, Knowledge systematization

## Abstract

**Background:**

One significant challenge in addressing the coronavirus disease 2019 (COVID-19) pandemic is to grasp a comprehensive picture of its infectious mechanisms. We urgently need a consistent framework to capture the intricacies of its complicated viral infectious processes and diverse symptoms.

**Results:**

We systematized COVID-19 infectious processes through an ontological approach and provided a unified description framework of causal relationships from the early infectious stage to severe clinical manifestations based on the homeostasis imbalance process ontology (HoIP). HoIP covers a broad range of processes in the body, ranging from normal to abnormal. Moreover, our imbalance model enabled us to distinguish viral functional demands from immune defense processes, thereby supporting the development of new drugs, and our research demonstrates how ontological reasoning contributes to the identification of patients at severe risk.

**Conclusions:**

The HoIP organises knowledge of COVID-19 infectious processes and related entities, such as molecules, drugs, and symptoms, with a consistent descriptive framework. HoIP is expected to harmonise the description of various heterogeneous processes and improve the interoperability of COVID-19 knowledge through the COVID-19 ontology harmonisation working group.

**Supplementary Information:**

The online version contains supplementary material available at 10.1186/s12911-024-02516-0.

## Background

The World Health Organization (WHO) declared severe acute respiratory syndrome coronavirus 2 (SARS-CoV2) in March 2020, and the number of infected individuals continues to increase worldwide. With the efforts of many researchers and the international community, knowledge about the coronavirus disease 2019 (COVID-19) is increasing and a large number of articles have been published. Additionally, several vaccines and drugs have been developed. However, because COVID-19 is highly complicated, understanding its underlying mechanisms remains challenging. One challenge is that COVID-19 can manifest as asymptomatic or life-threatening symptoms. In several case series with serial respiratory sampling, the peak viral load was observed immediately before or at the time of symptom onset [1]. Moreover, excessive inflammatory and immune responses in the lungs lead to acute respiratory distress syndrome (ARDS) [2].

To minimise the risk of progression to severe disease, technologies are needed to elucidate its mechanisms from diverse viewpoints, such as virology, immunology, molecular biology, clinical medicine, infectiology, pathology, and pharmacology.

Ontology is an attractive approach that contributes to explaining the basic elements underlying background comprehension and supports interdisciplinary knowledge systematisation.

This study proposes a homeostasis imbalance process ontology (HoIP) by focusing on homeostasis disturbances [3, 4]. Biological systems typically maintain a steady state and homeostasis. However, when homeostatic disturbances occur due to external factors such as viruses, various functional changes occur at several levels, from the cellular to the organ level. In this study, we first outlined the development of HoIP. Second, we describe the COVID-19 infectious processes and provide a unified representation framework in a consistent manner from the early phase of infection to severe clinical manifestations. We then illustrate the representation of various granularities in the COVID-19 infection process. Subsequently, we report an inferred analysis using ontology reasoning and discuss its contribution in reducing severe risks. HoIP contributes to the harmonisation of various COVID-19 ontologies to decrease the risk of COVID-19 and other new infectious diseases.

## Methods

We constructed HoIP using ontology editor Protégé 5.5.0. (https://protege.stanford.edu).

### Extension of TXPO

Yamagata et al. developed a toxic process ontology (TXPO) with a three-layer model from general to specialised toxicological terms [5]. Similar to TXPO, we designed HoIP with a three-layered structure. TXPO focuses only on liver toxicity courses and processes by drug (candidate)(s). We expanded these processes to fit general homeostatic imbalance courses including endoplasmic reticulum (ER) stress, mitochondrial damage at the organelle level, cell death at cell and tissue levels, and fatty liver at the organ level.

### Reuse of existing ontology

In the biomedical domain, HoIP reused the following existing ontologies: processes from Gene Ontology (GO) [6], anatomical entities from Uberon multi-species anatomy ontology (UBERON) [7], Cell Ontology (CL) [8], taxonomy information curated by the National Center for Biotechnology Information (NCBI) [9], compounds curated by Chemical Entities of Biological Interest (ChEBI) [10], proteins curated by Protein Ontology (PRO) [11], Phenotype and trait Ontology (PATO) [12] and Human Phenotype Ontology (HPO) [13], Symptom Ontology (http://www.obofoundry.org/ontology/symp.html), Disease Ontology (DO) [14], and Vaccine Ontology (VO) [15]. HoIP also uses Relation Ontology (RO) [16] to identify relations between entities. These entities were manually imported from the National Center for Biomedical Ontology (NCBO) BioPortal (https://bioportal.bioontology.org).

### Annotation and a new definition of the COVID-19 infectious process

**Fig 1:**
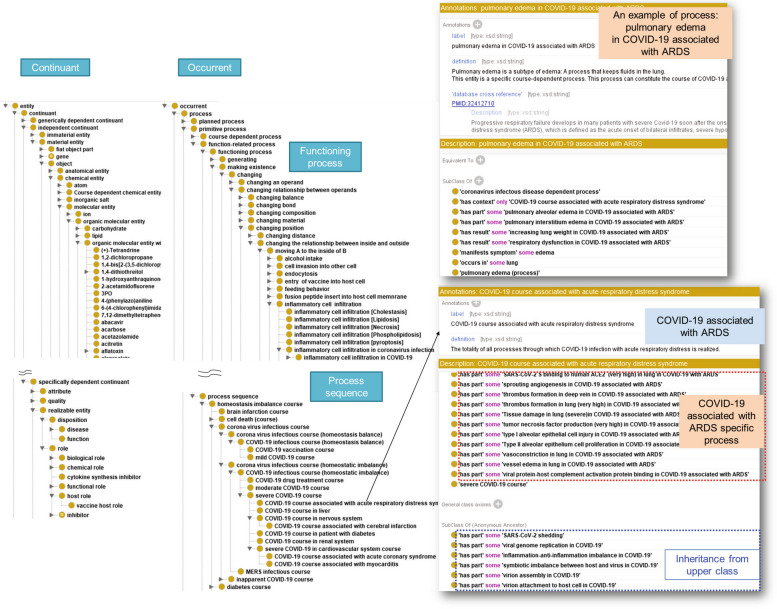
Overview of homeostasis imbalance process ontology (HoIP). Schematic representation of three-layer HoIP architecture Top layer: domain-neutral entities; intermediate layer: biomedicine-related entities; lower layer: homeostatic imbalance-related entities, including the COVID-19 infection course

The coronavirus and COVID-19 infectious processes were manually collected from textbooks on virology [17, 18], immunology [19], and medicine [20]. To objectively annotate and define entities according to well-established knowledge, we selected entities and their relationships described in textbooks. We also searched for COVID-19-related articles on PubMed using the MeSH terms: ‘COVID-19’, ‘SARS-CoV-2’, and ‘Severe acute respiratory syndrome-related coronavirus’. We have provided a list of the curated PubMed articles in Supplementary Information 1. All references for the entities were described using the annotation property ‘database cross reference’ (Fig. 1), and the relationships between entities are described by object properties. Each course describes its specific processes using a ‘has part’ relationship: For example, ‘HoIP: 0036006 COVID-19 associated with ARDS’ course ‘has part’ ‘HoIP: 0041811 thrombus formation in lung (very high)’ (Fig. 1 lower right). The processes were organised in an *is-a* (subclass of) hierarchy. For instance, ‘HoIP:0036025 increasing lung weight’ in COVID-19 associated with ARDS is generalised to ‘HoIP: 0000040 increasing weight’. Furthermore, the causal relationship between processes was described using a ‘has result’ relationship (for example, ‘HoIP: 0041840 pulmonary edema’ ‘has result’ ‘HoIP: 0036008 respiratory dysfunction’). We also annotated relationships between entities other than processes, such as ‘has participant’ for molecules/compounds, ‘occurs in’ for biological structures, ‘manifests symptom’ for symptoms, and ‘realises disease’ for diseases. The newly defined coronavirus and COVID-19 infection courses and their processes include drug treatment and vaccination. We have also added information on other disease-related processes (e.g. diabetes-related processes).

High-quality curation of biomedical knowledge is required to ensure HoIP data reliability. Therefore, in this study, manual annotation was employed instead of machine curation. In this study, expert biomedical curators with more than ten years of experience audited the data. Ontologists also checked for inheritance, definitions, and hierarchical structures.

New entities defined by HoIP use the prefix ‘HoIP_’. By inheriting the properties of the upper-layer terms, new terms are specialised to homeostasis-imbalance-specific terms and are defined at the lower level.

HoIP is formatted in Web Ontology Language (OWL). After editing, OWL-DL reasoner tools [21, 22] were used to check for logical consistency and identify contradictions. Inferences were made using the HermiT reasoner. HoIP is available from the NCBO BioPortal ontology repository site (https://bioportal.bioontology.org/ontologies/HOIP) and GitHub website (https://github.com/yuki-yamagata/hoip).

## Results

### Overview of HoIP

This section outlines the ontological structure of HoIP. We focused on the functioning of the human body, which is essential for maintaining homeostasis. To support the capture of a wide variety of infectious mechanisms, HoIP aims to cover entities from domain-neutral to COVID-19 domains with a three-layer structure:

The domain-neutral layer refers to the basic formal upper ontology (BFO) [23]. BFO supports the interoperation of different domain ontologies through consistent annotations. According to BFO, there is a dichotomy between the continuant and occurring entities of HoIP (Fig. 1). While occurrents contain the processes, continuants contain material entities that can preserve their identity even while gaining and losing material parts. Moreover, the lower classes are defined by inheriting the properties of the upper class and specialising in them while retaining their consistency. HoIP is characterised by the definition of general (biomedical-independent) functioning processes, such as increasing weight, transmitting, and transporting, with reference to functional ontology [24].

The intermediate layer of HoIP was organised by considering the reutilisation of the biomedical domain ontologies of the open community OBO Foundry [25]. Common biomedical processes were defined in the intermediate layer and did not include specific COVID-19 related entities.

The lower layer was created using defined entities related to homeostatic imbalances. The homeostasis imbalance course is defined as a series of changing processes in an organism from a homeostatic balance to an imbalance between functional supply and demand.

### Definition of COVID-19 infectious course

**Fig 2:**
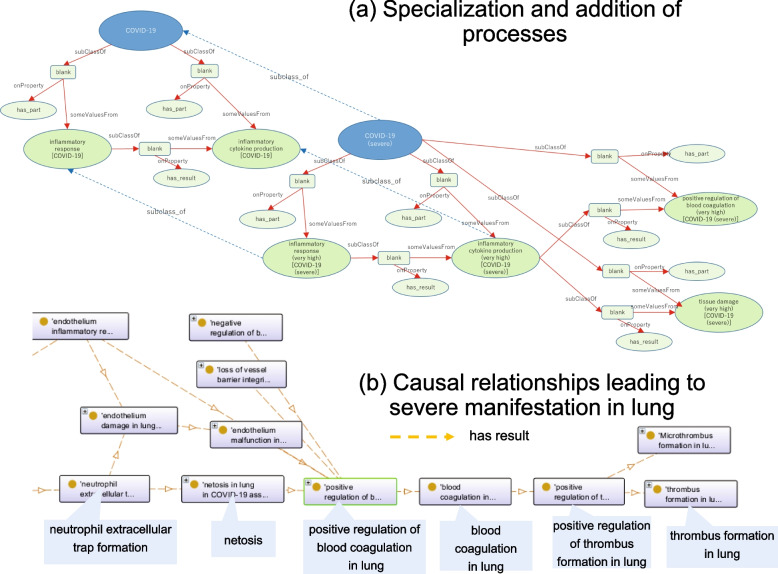
Examples of causal relationships between COVID-19 processes. Specialisation and adding processes: The subclasses of the COVID-19 course include severe COVID-19 (**a**) and the causal relationships of processes leading to severity, from ‘neutrophil extracellular trap formation’ to ‘thrombus formation in lung’ (**b**)

 In the lower layer, we defined the processes that constitute specific courses, including the course of COVID-19 (Fig. 1). This study defined the COVID-19 infectious course as ‘the totality of all processes involved in the infection to the homeostatic imbalance between the functional supply by the immune system and functional demand by SARS-CoV2. The course is represented by the causal relationships between processes. The subclasses of COVID-19, such as severe COVID-19, are defined by the specialisation of processes or the addition of new processes downstream (Fig. 2a). Furthermore, the subclasses of the severe COVID-19 course include COVID-19 associated with acute respiratory distress syndrome (ARDS). In COVID-19 associated with ARDS, various causal relationships between the processes leading to the progression of lung disease severity have been described. An example of the path is ‘neutrophil extracellular trap (NET) formation’ can cause ‘netosis in lung’, leading to ‘positive regulation of blood coagulation in lung’ and ‘blood coagulation in lung (very high). Consequently, positive regulation of thrombus formation in the lungs can occur (Fig. 2b).

We confirmed that the HoIP ontology can show commonalities and differences in multiple courses using this three-layer structure. For example, inflammatory cell infiltration into the liver can occur during the course of fatty liver disease, whereas inflammatory cell infiltration into the lungs can occur in COVID-19 associated with ARDS, although both have the same upper class of ‘inflammatory cell infiltration’. As another example, the HoIP hierarchical tree indicates that microvascular dysfunction in diabetes and COVID-19 share a common upper class of ‘microvascular dysfunction’. Moreover, HoIP describes the former causes ‘renal damage,’ and the latter causes ‘thrombus formation in the lung’ using the ‘has result’ object property.

### Representation of COVID-19 infectious processes based on a homeostatic imbalance

Because viruses cannot replicate themselves and require host cell replication mechanisms, their effects on the host can be highly variable. In this study, we represent viral infection mechanisms as a type of homeostatic imbalance triggered by viruses. Thus, we introduce the imbalance theory [26] and describe a unified framework that comprises: 1) the viral functional demand process, 2) the biological defense functioning process of the immune system, 3) the balance/imbalance between them, and 4) the establishment of viral infection as an outcome. To indicate the functional performance level, we introduce the following degree values: ‘very low’, ‘low’, ‘moderate’, ‘high’, and ‘very high’. Under normal conditions, we set the functional level as ‘moderate’. In our model, inflammation is represented by the immune defense against viral action. Using our framework, we present the representation of COVID-19 courses as follows.

- Mild COVID-19 infectious course: If viral exposure is not high and functional demand is ‘moderate’, the immune system enables ‘moderate’ work, and the balance between viral functional demand and immune defense can be maintained. Therefore, the virus was eliminated. During the mild course, inflammatory responses can cause fever, swelling, redness, and pain, similar to those of the common cold.
- COVID-19 infection course with vaccination: Upon vaccination, if the virus invades, the immune defense works without inhibiting immune responses. Thus, the balance between the functional demand of the virus and the immune defense function is maintained, and the virus is eliminated. However, vaccination may induce inflammatory responses involving adverse reactions of immune defense, such as fever, swelling, and pain. In rare cases, thrombus formation and inflammatory response in the myocardium associated with myocarditis due to high levels of immune responses might occur.
- Inapparent COVID-19 infectious course: The immune system works at a lower (low) level than the normal range due to the inhibition of interferons by the virus. An imbalance between viral functional demands and immune defence functions occurs, and the establishment of a viral infection can occur as an outcome. However, since the inflammatory response resulting from the immune defense is weak, no symptoms are observed.
- Severe COVID-19 infectious course: The inflammatory response works as ‘very high’, resulting from excessive immune defense. In the lungs, the infiltration of inflammatory cells resulting from the immune response causes alveolar epithelial tissue injury and oedema, leading to hyaline membrane formation and respiratory dysfunction. Furthermore, due to the hyperproduction of inflammatory cytokines, adjacent cell injury of infected cells by inflammatory cells may occur, leading to systemic inflammation and multiorgan dysfunction.

Thus, we confirmed that our framework can represent various infectious courses from before manifestation to severity with flexibility, explaining the inflammation resulting from immune defences against SARS-CoV-2.

### Representation of various granularities of COVID-19 infectious processes

**Fig 3:**
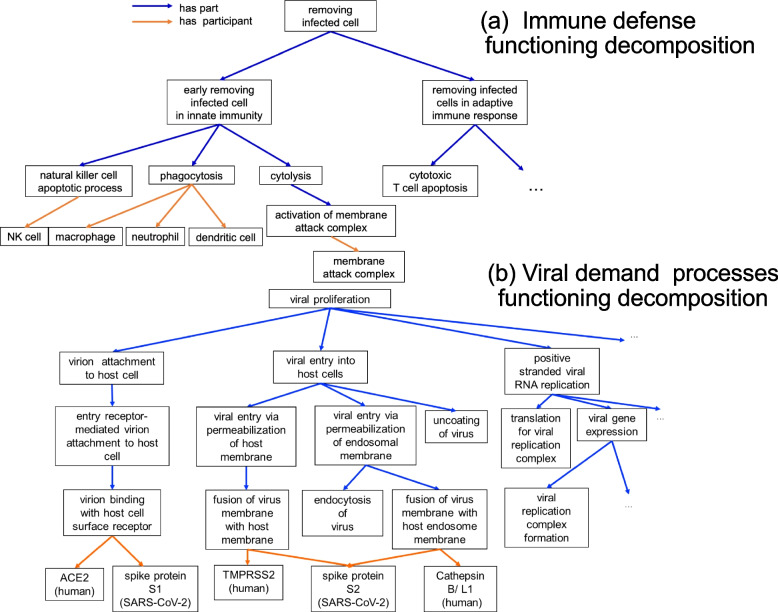
An example of functional decomposition. Examples of the decomposing functioning processes are presented. **a** ‘removing infected cell’ as a biological defense functioning. **b** ‘viral proliferation’ as a viral demand process

In HoIP, each infectious course has its specific processes using ‘has part’/‘part of’ relationships. Furthermore, biological systems typically function with multiple granularities. Therefore, based on the functional modelling approach [24], we refined the functioning process of the biological system by decomposing it into subfunctioning processes. For instance, the removal of infected cells is a function of biological defense (Fig. 3a). Thus, to achieve this function, ‘early removing infected cell in innate immunity’ and ‘removing infected cell in adaptive immune response’ work as sub-functioning processes. Concerning ‘early removing infected cell in innate immunity’, the process is further decomposed into the following three sub-functioning processes: ‘phagocytosis’, ‘natural killer cell apoptotic process’, and ‘cytolysis’. Macrophage, neutrophil, and dendritic cells are described as participants of phagocytosis by using the ‘has participant’ relationship. During the COVID-19 (severe) course, ‘removing infected cell’ has ‘netosis’ as a sub-functioning process, which causes ‘vascular endothelial cell damage’ and ‘positive regulation of blood coagulation’. Similarly, we confirmed that the viral demand processes could be decomposed (Fig. 3b).

### Related entities of COVID-19 infectious processes

**Fig 4:**
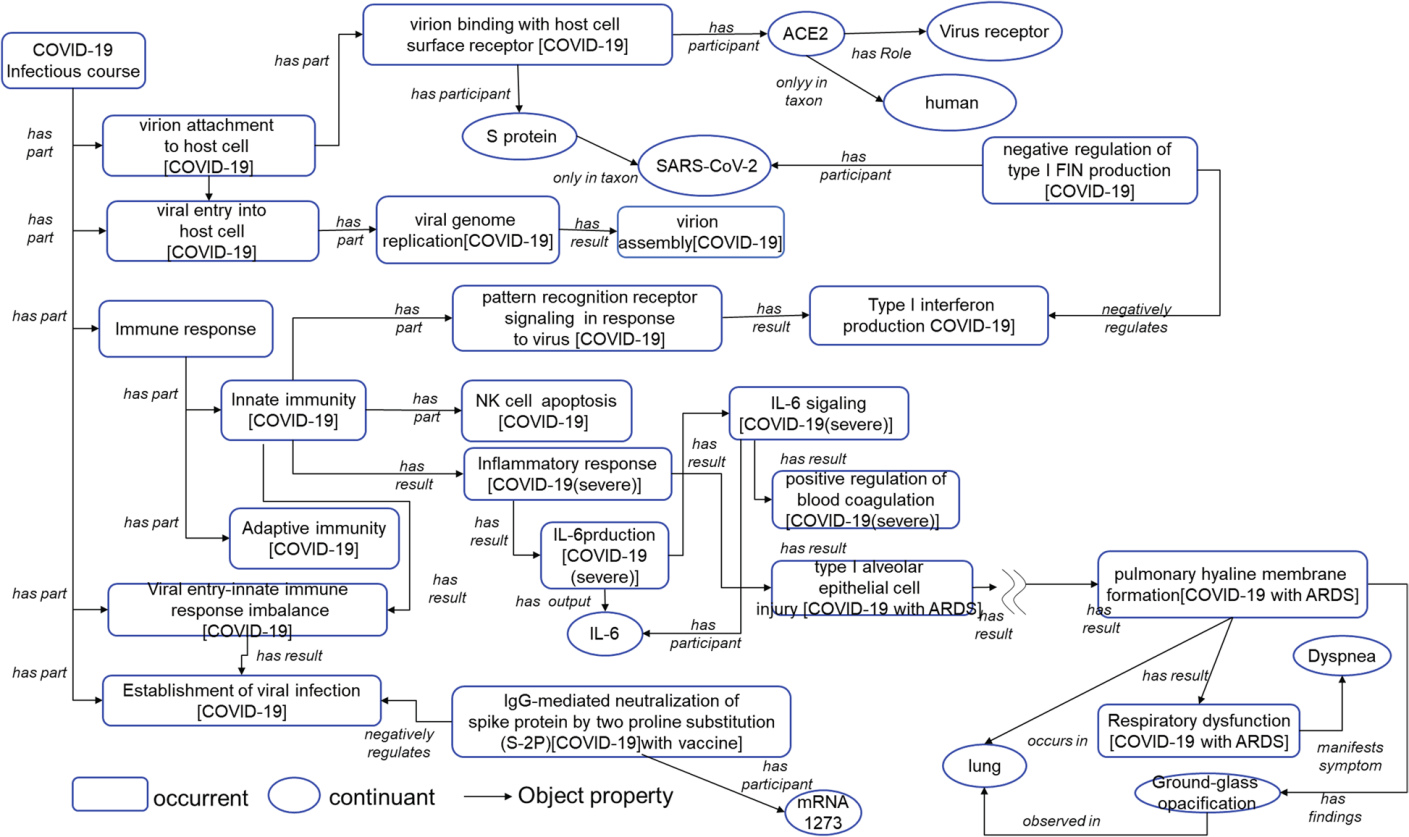
Relationships between entities in COVID-19 infectious course. This figure illustrates the ontological design patterns for the COVID-19 pandemic. The rounded rectangle shows the processes, and the ellipses indicate the continuants

To better understand infectious mechanisms, it is crucial to understand how these processes are related to other entities. Fig. 4 shows an example of the relationship between the COVID-19 entities. In this study, molecules involved in each process are described by using the ‘has participant’ relation. For instance, ACE2 is known to be involved in the renin-angiotensin system (RAS) in living organisms under normal conditions, whereas in the COVID-19 infectious course in HoIP, we can see that ACE2 participates in the ‘virion binding with host cell surface receptor’ and plays a ‘virus receptor’ role. Furthermore, it is essential to determine whether each molecule is derived from a virus or a host. Using the NCBI Taxonomy DB and ‘only in taxon’ relation, for example, HoIP shows that the ACE2 molecule was derived from humans, whereas the S protein is a molecule derived from SARS-CoV-2.

In addition to the biological molecules, we annotated the target processes of COVID-19 vaccines and drugs in the body. As a result, we confirmed that many vaccines are related to ‘immunoglobulin-mediated neutralisation of S protein’ by using the ‘has participant’ relation. In addition, mRNA-1273 is a participant in ‘IgG-mediated neutralisation of spike protein by two proline substitutions (S-2P)’.

To reduce severity risks, we should identify where the infectious process occurs; ‘occurs in’ relationship is used in the ontology; an example is pulmonary hyaline membrane formation ‘occurs in’ the lung.

Concerning diagnosis, we defined clinical findings, which are subtypes of data, using ‘observed in’ relationships such as ‘Ground glass opacification on pulmonary HRCT’ observed in the lung. HoIP explicates the relationships between processes and symptoms, such as the process of vascular hyperpermeability and the symptoms of redness, by using the ‘manifests phenotype’ relation. An example of severity is the leakage of fluid into the alveolar space leading to pulmonary oedema, which causes respiratory dysfunction, with dyspnoea as a severe manifestation.

### Finding severity risks by reasoning

HoIP causes a wide variety of homeostatic imbalances. To identify the potential underlying diseases that affect severe clinical manifestations, we analysed causal relationships across multiple courses of HoIP. HoIP is described in the OWL DL ontology based on description logic. In this study, ‘has result’ representing the causal relationships of processes is logically defined as transitive (owl: Transitive Property), whereas ‘has cause’ is the inverse (owl: inverseOf).

**Fig 5:**
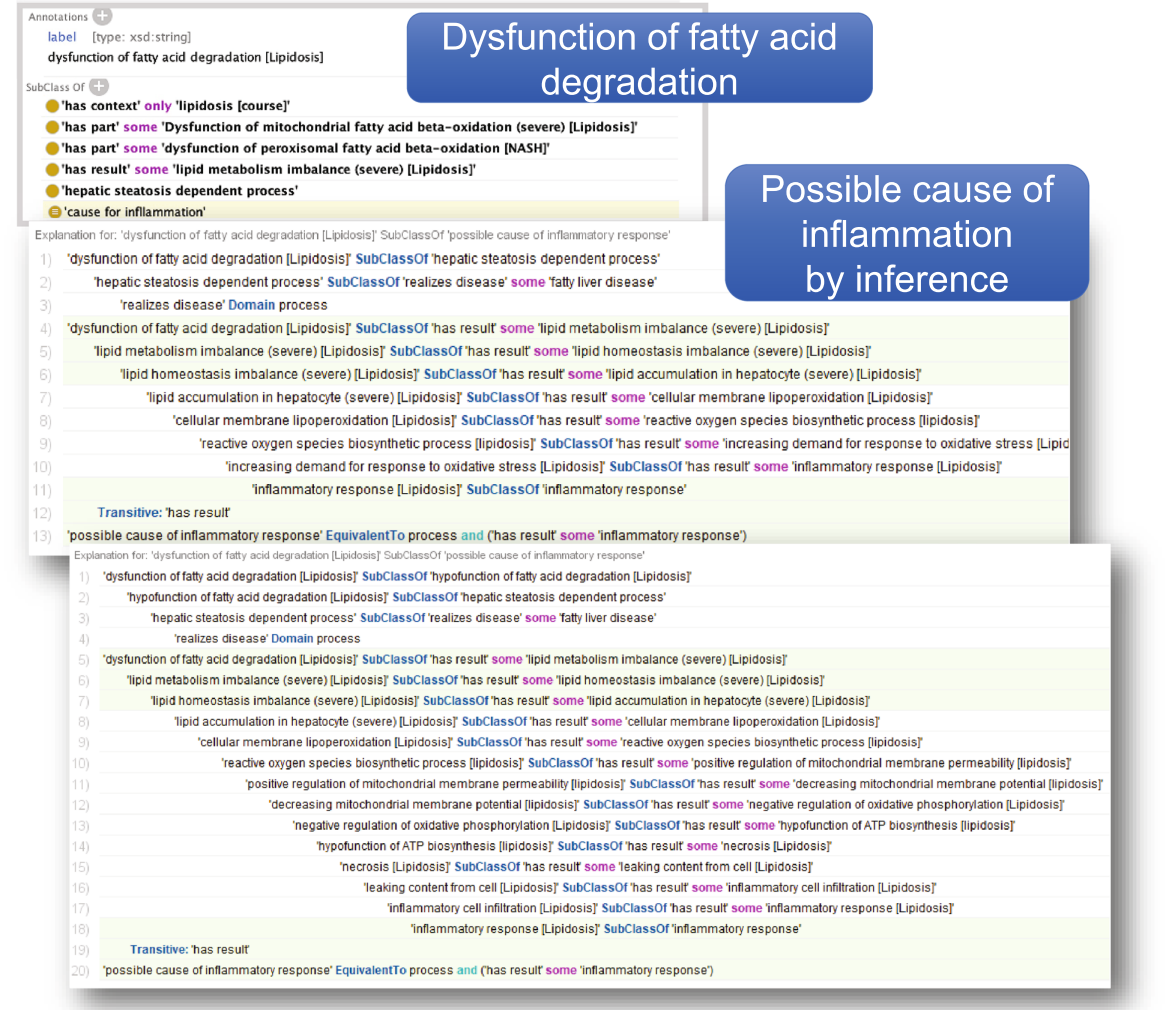
Inference examples of upstream inflammatory response across multiple homeostatic imbalance courses. Using the HermiT reasoner in the Protégé tool, dysfunction of fatty acid degradation, as an example of the cause of the inflammatory response, was observed. One inference shows that dysfunction of fatty acid degradation in lipidosis can lead to inflammation via the ROS biosynthetic process. Other studies have shown that ROS production can cause positive regulation of mitochondrial membrane permeability and hypofunction during ATP biosynthesis, leading to the inflammatory response

The inflammatory response is a vital process for predicting severity. To identify the causal processes, we inferred the upstream inflammatory response using the HermiT reasoner. The results are shown in Fig. 5. We found that the ‘dysfunction of fatty acid degradation’ in the fatty liver involves several pathways, which can lead to inflammation. Most of these mediate ‘reactive oxygen species (ROS) biosynthetic process’.

**Fig 6:**
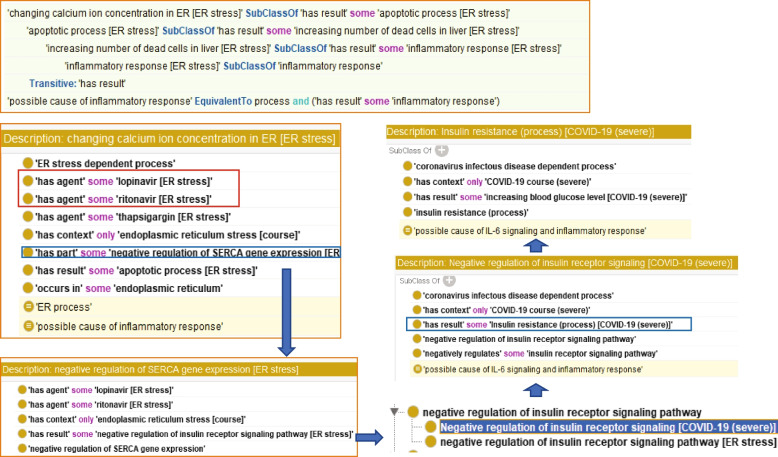
Inference example of finding a risk of the drugs. This inference (upper panel) indicates that a change in the calcium ion concentration in ER stress can cause inflammation. The ontological description shows ‘Negative regulation of SERCA gene expression,’ the subprocess of the ‘Changing calcium ion in ER stress’ has agents lopinavir and ritonavir (middle and lower left). The causal relationship shows that this sub-process can cause negative regulation of the insulin receptor pathway, which is common to the COVID-19 pathway (lower right panel). Furthermore, it led to insulin resistance in COVID-19 patients (middle right panel)

**Fig 7:**
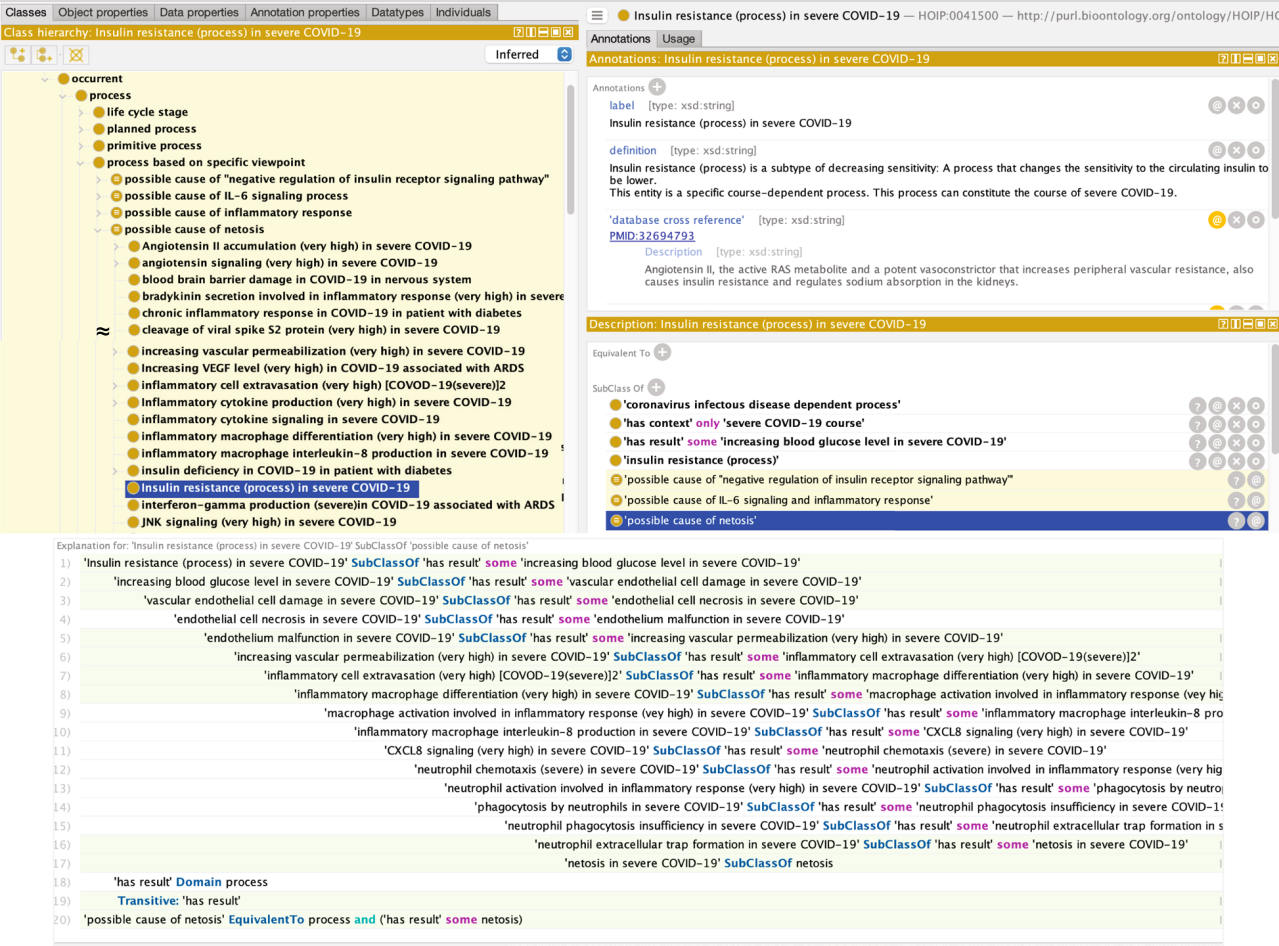
Inference example of insulin resistance to netosis. Inference shows that Insulin resistance in severe COVID-19 can ‘increasing blood glucose level’ (hyperglycaemia), causing ‘vascular endothelial cell damage’, ‘neutrophil extracellular trap formation’, and ‘necrosis’

Across multiple courses, we also found that lopinavir and ritonavir, known COVID-19 drugs, participate in changing calcium ion concentrations during ER stress, which can also lead to inflammation. Moreover, the sub-process ‘negative regulation of SERCA gene expression’ can cause ‘negative regulation of insulin receptor signalling pathway’. In the HoIP *is-a* hierarchy, negative regulation of the insulin receptor signalling pathway is common in severe COVID-19. In severe COVID-19, the ‘negative regulation of the insulin receptor pathway, inferred as a possible cause of IL-6 signalling and inflammatory responses, can lead to ‘insulin resistance’ and ‘increasing blood glucose level’ (Fig. 6). Further inference results also demonstrated that insulin resistance in COVID-19 patients could lead to NET formation and netosis (Fig. 7).

## Discussion

Understanding the mechanisms of COVID-19 is challenging because the relevant processes in the host body are highly diverse. Several databases, such as the disease map [27] and Kyoto Encyclopedia of Genes and Genomes (KEGG) [28], provide molecular pathways in COVID-19; however, little is known about cell-to-organ-level processes. As a theoretical contribution, HoIP, by introducing the functional decomposition approach, enables the coverage of processes across granularities in COVID-19 from the molecular to the organ level, making it computer-processable. Therefore, our approach supports a comprehensive understanding of how the immune system achieves the entire defense function at the macro level and how various immune cells function in sub-functional processes.

Based on the perspective of homeostatic disturbances, our approach provides a flexible representation framework with consistency, covering the early stages of infection and mild-to-severe manifestations of COVID-19, such as respiratory failure. Our representation framework shows how inflammation varies depending on the degree of immune defense against viral infections, and leads to disease severity. Therefore, our model has practical applications and may help optimise the timing of anti-inflammatory drug treatment.

As a practical implication, the analysis of the causal relationships of processes in COVID-19 courses will be helpful for risk management. Comparing the downstream causal relationships of the processes between the severe COVID-19 course and the inapparent or mild ones will facilitate severity risk management in the early phase of infection. In addition, concerning the diagnosis, an upstream-focused analysis supports the identification of causal factors.

The key point of our approach is that HoIP can provide rich information on various processes across multiple mechanisms, ranging from basic physiological phenomena to medicine. Identifying the potential risk of COVID-19 in patients with chronic diseases is challenging. Analysing the general causal relationships between processes across multiple courses using HoIP helps identify potential risks. In this study, the ontological inference of possible causes of inflammation demonstrated that multiple crosstalks exist between the course of COVID-19 and fatty liver disease. Multiple pathways involved in the dysfunction of fatty acid degradation-mediated ROS production, that is, oxidative stress, explain why patients with fatty liver develop severe disease. Some studies have reported that non-alcoholic fatty liver disease (NAFLD) is associated with a high risk of severity in COVID-19 patients [29]. Our approach will contribute to the planning and management of NAFLD and non-alcoholic steatohepatitis (NASH).

Moreover, from another inference result, the drugs lopinavir and ritonavir might cause insulin resistance in COVID-19 via negative regulation of the insulin receptor pathway during ER stress. Interestingly, ‘insulin resistance’ in COVID-19 could lead to both ‘IL-6 signalling and inflammation’ and ‘NET formation’. As shown in Fig. 2, the latter may positively regulate thrombus formation in the lungs. Therefore, we will further investigate the relationship between ER stress, COVID-19, and diabetes and the adverse effects of these drugs for severe risk management.

By suggesting general causal relationships between processes across multiple courses, HoIP enables us to integrate the fragmented knowledge of various mechanisms, bridge gaps between basic and clinical medicine, and fill in missing links.

The three-layered structure of HoIP enables us to capture an entity’s intrinsic nature with inheritance considerations, that is, vertical relationships. In addition, the representation of the causal relationships of processes explains the horizontal relationships. Thus, our approach helps clarify the complete picture of the severity mechanisms of COVID-19.

We have now begun international cooperation and aligned it with multiple ontologies, such as the Coronavirus Infectious Disease Ontology (CIDO) [30], by creating a COVID-19 ontology harmonisation working group for interoperability [31]. HoIP is expected to provide consistent knowledge of the causal relationships between biological processes during the early period of infection, the asymptomatic period (latency period), and disease onset. The CIDO has a rich information system for anti-coronavirus drugs and drug-target networks. The ontology for the collection and analysis of COVID-19 data (CODO) [32] includes statistical data on disease spread and casualties by space and time. By integrating and extending the knowledge of various ontologies, we can challenge the support of new drugs and decrease the risk of COVID-19 and newly emerging infectious diseases.

Our study was limited in that we did not cover the many side effects or toxic processes associated with COVID-19 drugs and vaccines. Every new drug has the potential risk of unexpected adverse events. In our future work on COVID-19, we plan to enhance our knowledge by annotating toxic processes using TXPO. This study provides helpful information to support the safe management of COVID-19 drug development and drug repositioning. Another limitation of this study is its usability. Although manual annotation provides high-quality knowledge systematisation, we did not cover the latest information. Therefore, we will consider semi-automatic annotations in the future. By combining annotation by human experts with natural language processing (NLP) and machine learning techniques for updates, the ontology can incorporate new research findings in a timely manner. Large Language Models (LLMs) are also helpful for annotation (for example, in identifying molecules and drugs in articles).

In future studies, we intend to enhance the practical significance by incorporating data sources beyond textbooks and articles, such as real-time patient data and epidemiological studies. These additions enhance the depth and relevance of the ontology, making it more applicable and aligned with real-world clinical scenarios. We will explore the feasibility and benefits of integrating HoIP with medical databases and electronic health records (EHR) to improve clinical utility, including early treatment planning for risk management.

## Conclusion

We developed a new ontology, HoIP, covering COVID-19 infectious processes and related body structures, molecules, symptoms, and diseases. This will support the understanding of the mechanisms of the COVID-19 infectious process from mild to severe manifestations and will help optimise treatment management to decrease severity risks.

### Supplementary Information

**Additional file 1.** PubMed article list for COVID-19 infectious disease process annotation

## Data Availability

The ontology data file is available on the NCBO BioPortal ontology repository site at https://bioportal.bioontology.org/ontologies/HOIP. The data is also available on the GitHub website at https://github.com/yuki-yamagata/hoip.

## Abbreviations

ARD: Acute Respiratory Distress Syndrome
BFO: Basic Formal Ontology
ChEBI: Chemical Entities of Biological Interest
CIDO: Coronavirus Infectious Disease Ontology
CL: Cell Ontology
CODO: COviD-19 data
COVID-19: Coronavirus Disease 2019
DO: Disease Ontology
ER: endoplasmic reticulum
GO: Gene Ontology
HoIP: homeostasis imbalance process
HP: Human Phenotype Ontology
NAFLD: non-alcoholic fatty liver diseases
NASH: non-alcoholic steatohepatitis
NCBI: National Center for Biotechnology Information
NCBO: National Center for Biomedical Ontology
NET: neutrophil extracellular trap
OWL: Ontology Web Language
PATO: Phenotype and trait ontology
PRO: Protein Ontology
RAS: Renin-Angiotensin System
RO: Relation Ontology
ROS: Reactive Oxygen Species
SARS-CoV2: severe acute respiratory syndrome coronavirus 2
TXPO: toxic process ontology
UBERON: Uberon multi-species anatomy ontology
VO: Vaccine Ontology
WHO: World Health Organization
