# Supplementary material for: Homeostasis imbalance process ontology: a study on COVID-19 infectious processes
Source: BMC Med Inform Decis Mak. 2024 May 22;23(Suppl 4):301. doi: 10.1186/s12911-024-02516-0 (PMC11110177; doi:10.1186/s12911-024-02516-0)
Supplement: Supplementary file 1 — Additional file 1. PubMed article list for COVID-19 infectious disease process annotation [file 12911_2024_2516_MOESM1_ESM.pdf]

Title

Homeostasis Imbalance Process Ontology: a Study on COVID-19 Infectious Processes

Authors

Yuki Yamagata[1,2,§], Tatsuya Kushida[3], a, and Shuichi Onami[1,2] and Hiroshi

Masuya[1,3]

Institutions and Addresses

[1]Life Science Data Sharing Unit, Infrastructure Research and Development Division, RIKEN Information R&D and Strategy Headquarters, 2-2-3 Minatojima-minamimachi, Chuo-ku, Kobe, Hyogo 650-0047, Japan

[2]Laboratory for Developmental Dynamics, RIKEN Center for Biosystems Dynamics Research, 2-2-3 Minatojima-minamimachi, Chuo-ku, Kobe, Hyogo 650-0047, Japan

[3]Integrated Bioresource Information Division, RIKEN BioResource Research Center, 3-1-1 Koyadai, Tsukuba-shi, Ibaraki 305-0074, Japan

Correspondence:

Yuki Yamagata (E-mail: yuki.yamagata@riken.jp)

Supplementary Information 1

PubMed article list for COVID-19 infectious disease process annotation

Supplementary Information 1. PubMed article list for COVID-19 infectious disease process annotation

1: Liu X, Zhao J, Wang H, Wang W, Su X, Liao X, Zhang S, Sun J, Zhang Z. Metabolic Defects of Peripheral T Cells in COVID-19 Patients. J Immunol. 2021 Jun 15;206(12):2900-2908. doi: 10.4049/jimmunol.2100068. Epub 2021 May 28. PMID: 34049969.

2: Zawawi A, Naser AY, Alwafi H, Minshawi F. Profile of Circulatory Cytokines and Chemokines in Human Coronaviruses: A Systematic Review and Meta-Analysis. Front Immunol. 2021 May 5;12:666223. doi: 10.3389/fimmu.2021.666223. PMID: 34046036; PMCID: PMC8147689.

3: Gritti G, Raimondi F, Bottazzi B, Ripamonti D, Riva I, Landi F, Alborghetti L, Frigeni M, Damiani M, Micò C, Fagiuoli S, Lorini FL, Gandini L, Novelli L, Morgan JP, Owens BMJ, Kanhai KJK, Reljanovic GT, Rizzi M, Di Marco F, Mantovani A, Rambaldi A. Siltuximab downregulates interleukin-8 and pentraxin 3 to improve ventilatory status and survival in severe COVID-19. Leukemia. 2021 Sep;35(9):2710-2714. doi: 10.1038/s41375-021-01299-x. Epub 2021 May 24. Erratum in: Leukemia. 2021 Jun 28;: PMID: 34031531; PMCID: PMC8142063.

4: Pashaei Y. Drug repurposing of selective serotonin reuptake inhibitors: Could these drugs help fight COVID-19 and save lives? J Clin Neurosci. 2021 Jun;88:163-172. doi: 10.1016/j.jocn.2021.03.010. Epub 2021 Mar 19. PMID: 33992179; PMCID: PMC7973060.

5: Marco-Martínez J, Elola-Somoza FJ, Fernández-Pérez C, Bernal-Sobrino JL, Azaña-Gómez FJ, García-Klepizg JL, Andrès E, Zapatero-Gaviria A, Barba-Martin R, Calvo-Manuel E, Canora-Lebrato J, Lorenzo-Villalba N, Méndez-Bailón M. Heart Failure Is a Poor Prognosis Risk Factor in Patients Undergoing Cholecystectomy: Results from a Spanish Data-Based Analysis. J Clin Med. 2021 Apr 16;10(8):1731. doi: 10.3390/jcm10081731. PMID: 33923710; PMCID: PMC8072897.

6: Kennedy NA, Goodhand JR, Bewshea C, Nice R, Chee D, Lin S, Chanchlani N, Butterworth J, Cooney R, Croft NM, Hart AL, Irving PM, Kok KB, Lamb CA, Limdi JK, Macdonald J, McGovern DP, Mehta SJ, Murray CD, Patel KV, Pollok RC, Raine T, Russell RK, Selinger CP, Smith PJ, Bowden J, McDonald TJ, Lees CW, Sebastian S, Powell N, Ahmad T; Contributors to the CLARITY IBD study. Anti-SARS-CoV-2 antibody responses are attenuated in patients with IBD treated with infliximab. Gut. 2021 May;70(5):865-875. doi: 10.1136/gutjnl-2021-324388. Epub 2021 Mar 22. PMID: 33753421; PMCID: PMC7992387.

7: Carvalho T, Krammer F, Iwasaki A. The first 12 months of COVID-19: a timeline of immunological insights. Nat Rev Immunol. 2021 Apr;21(4):245-256. doi: 10.1038/s41577-021-00522-1. Epub 2021 Mar 15. PMID: 33723416; PMCID: PMC7958099.

8: Teijaro JR, Farber DL. COVID-19 vaccines: modes of immune activation and future challenges. Nat Rev Immunol. 2021 Apr;21(4):195-197. doi: 10.1038/s41577-021-00526-x. PMID: 33674759; PMCID: PMC7934118.

9: Higuera-de la Tijera F, Servín-Caamaño A, Reyes-Herrera D, Flores-López A,

Robiou-Vivero EJA, Martínez-Rivera F, Galindo-Hernández V, Chapa-Azuela O, Chávez-Morales A, Rosales-Salyano VH. Impact of liver enzymes on SARS-CoV-2 infection and the severity of clinical course of COVID-19. Liver Res. 2021 Mar;5(1):21-27. doi: 10.1016/j.livres.2021.01.001. Epub 2021 Jan 12. PMID: 33520337; PMCID: PMC7831761.

10: Zhao Z, Qin P, Huang YW. Lysosomal ion channels involved in cellular entry and uncoating of enveloped viruses: Implications for therapeutic strategies against SARS-CoV-2. Cell Calcium. 2021 Mar;94:102360. doi: 10.1016/j.ceca.2021.102360. Epub 2021 Jan 23. PMID: 33516131; PMCID: PMC7825922.

11: Kyrou I, Randeva HS, Spandidos DA, Karteris E. Not only ACE2-the quest for additional host cell mediators of SARS-CoV-2 infection: Neuropilin-1 (NRP1) as a novel SARS-CoV-2 host cell entry mediator implicated in COVID-19. Signal Transduct Target Ther. 2021 Jan 18;6(1):21. doi: 10.1038/s41392-020-00460-9. PMID: 33462185; PMCID: PMC7812344.

12: Carsetti R, Zaffina S, Piano Mortari E, Terreri S, Corrente F, Capponi C, Palomba P, Mirabella M, Cascioli S, Palange P, Cuccaro I, Milito C, Zumla A, Maeurer M, Camisa V, Vinci MR, Santoro A, Cimini E, Marchioni L, Nicastri E, Palmieri F, Agrati C, Ippolito G, Porzio O, Concato C, Onetti Muda A, Raponi M, Quintarelli C, Quinti I, Locatelli F. Different Innate and Adaptive Immune Responses to SARS-CoV-2 Infection of Asymptomatic, Mild, and Severe Cases. Front Immunol. 2020 Dec 16;11:610300. doi: 10.3389/fimmu.2020.610300. PMID: 33391280; PMCID: PMC7772470.

13: Dai L, Gao GF. Viral targets for vaccines against COVID-19. Nat Rev Immunol. 2021 Feb;21(2):73-82. doi: 10.1038/s41577-020-00480-0. Epub 2020 Dec 18. PMID: 33340022; PMCID: PMC7747004.

14: Widge AT, Rouphael NG, Jackson LA, Anderson EJ, Roberts PC, Makhene M, Chappell JD, Denison MR, Stevens LJ, Pruijssers AJ, McDermott AB, Flach B, Lin BC, Doria-Rose NA, O'Dell S, Schmidt SD, Neuzil KM, Bennett H, Leav B, Makowski M, Albert J, Cross K, Edara VV, Floyd K, Suthar MS, Buchanan W, Luke CJ, Ledgerwood JE, Mascola JR, Graham BS, Beigel JH; mRNA-1273 Study Group. Durability of Responses after SARS-CoV-2 mRNA-1273 Vaccination. N Engl J Med. 2021 Jan 7;384(1):80-82. doi: 10.1056/NEJMc2032195. Epub 2020 Dec 3. PMID: 33270381; PMCID: PMC7727324.

15: Alon R, Sportiello M, Kozlovski S, Kumar A, Reilly EC, Zarbock A, Garbi N, Topham DJ. Leukocyte trafficking to the lungs and beyond: lessons from influenza for COVID-19. Nat Rev Immunol. 2021 Jan;21(1):49-64. doi: 10.1038/s41577-020-00470-2. Epub 2020 Nov 19. PMID: 33214719; PMCID: PMC7675406.

16: Gu SX, Tyagi T, Jain K, Gu VW, Lee SH, Hwa JM, Kwan JM, Krause DS, Lee AI, Halene S, Martin KA, Chun HJ, Hwa J. Thrombocytopathy and endotheliopathy: crucial contributors to COVID-19 thromboinflammation. Nat Rev Cardiol. 2021 Mar;18(3):194-209. doi: 10.1038/s41569-020-00469-1. Epub 2020 Nov 19. PMID: 33214651; PMCID: PMC7675396.

17: Lim S, Bae JH, Kwon HS, Nauck MA. COVID-19 and diabetes mellitus: from

pathophysiology to clinical management. Nat Rev Endocrinol. 2021 Jan;17(1):11-30. doi: 10.1038/s41574-020-00435-4. Epub 2020 Nov 13. PMID: 33188364; PMCID: PMC7664589.

18: Lee S, Channappanavar R, Kanneganti TD. Coronaviruses: Innate Immunity, Inflammasome Activation, Inflammatory Cell Death, and Cytokines. Trends Immunol. 2020 Dec;41(12):1083-1099. doi: 10.1016/j.it.2020.10.005. Epub 2020 Oct 15. PMID: 33153908; PMCID: PMC7561287.

19: Flanagan KL, Best E, Crawford NW, Giles M, Koirala A, Macartney K, Russell F, Teh BW, Wen SC. Progress and Pitfalls in the Quest for Effective SARS-CoV-2 (COVID-19) Vaccines. Front Immunol. 2020 Oct 2;11:579250. doi: 10.3389/fimmu.2020.579250. PMID: 33123165; PMCID: PMC7566192.

20: V'kovski P, Kratzel A, Steiner S, Stalder H, Thiel V. Coronavirus biology and replication: implications for SARS-CoV-2. Nat Rev Microbiol. 2021 Mar;19(3):155-170. doi: 10.1038/s41579-020-00468-6. Epub 2020 Oct 28. PMID: 33116300; PMCID: PMC7592455.

21: Perico L, Benigni A, Casiraghi F, Ng LFP, Renia L, Remuzzi G. Immunity, endothelial injury and complement-induced coagulopathy in COVID-19. Nat Rev Nephrol. 2021 Jan;17(1):46-64. doi: 10.1038/s41581-020-00357-4. Epub 2020 Oct 19. PMID: 33077917; PMCID: PMC7570423.

22: Walsh EE, Frenck RW Jr, Falsey AR, Kitchin N, Absalon J, Gurtman A, Lockhart S, Neuzil K, Mulligan MJ, Bailey R, Swanson KA, Li P, Koury K, Kalina W, Cooper D, Fontes-Garfias C, Shi PY, Türeci Ö, Tompkins KR, Lyke KE, Raabe V, Dormitzer PR, Jansen KU, Şahin U, Gruber WC. Safety and Immunogenicity of Two RNA-Based Covid-19 Vaccine Candidates. N Engl J Med. 2020 Dec 17;383(25):2439-2450. doi: 10.1056/NEJMoa2027906. Epub 2020 Oct 14. PMID: 33053279; PMCID: PMC7583697.

23: Sahin U, Muik A, Derhovanessian E, Vogler I, Kranz LM, Vormehr M, Baum A, Pascal K, Quandt J, Maurus D, Brachtendorf S, Lörks V, Sikorski J, Hilker R, Becker D, Eller AK, Grützner J, Boesler C, Rosenbaum C, Kühnle MC, Luxemburger U, Kemmer-Brück A, Langer D, Bexon M, Bolte S, Karikó K, Palanche T, Fischer B, Schultz A, Shi PY, Fontes-Garfias C, Perez JL, Swanson KA, Loschko J, Scully IL, Cutler M, Kalina W, Kyratsous CA, Cooper D, Dormitzer PR, Jansen KU, Türeci Ö. COVID-19 vaccine BNT162b1 elicits human antibody and T_H1 T cell responses. Nature. 2020 Oct;586(7830):594-599. doi: 10.1038/s41586-020-2814-7. Epub 2020 Sep 30. Erratum in: Nature. 2021 Feb;590(7844):E17. PMID: 32998157.

24: Krammer F. SARS-CoV-2 vaccines in development. Nature. 2020 Oct;586(7830):516-527. doi: 10.1038/s41586-020-2798-3. Epub 2020 Sep 23. PMID: 32967006.

25: Jeyanathan M, Afkhami S, Smaill F, Miller MS, Lichty BD, Xing Z. Immunological considerations for COVID-19 vaccine strategies. Nat Rev Immunol. 2020 Oct;20(10):615-632. doi: 10.1038/s41577-020-00434-6. Epub 2020 Sep 4. PMID: 32887954; PMCID: PMC7472682.

26: De Virgiliis F, Di Giovanni S. Lung innervation in the eye of a cytokine

storm: neuroimmune interactions and COVID-19. Nat Rev Neurol. 2020 Nov;16(11):645-652. doi: 10.1038/s41582-020-0402-y. Epub 2020 Aug 25. PMID: 32843733; PMCID: PMC7446605.

27: McGonagle D, O'Donnell JS, Sharif K, Emery P, Bridgewood C. Immune mechanisms of pulmonary intravascular coagulopathy in COVID-19 pneumonia. Lancet Rheumatol. 2020 Jul;2(7):e437-e445. doi: 10.1016/S2665-9913(20)30121-1. Epub 2020 May 7. PMID: 32835247; PMCID: PMC7252093.

28: Magoon S, Bichu P, Malhotra V, Alhashimi F, Hu Y, Khanna S, Berhanu K. COVID-19-Related Glomerulopathy: A Report of 2 Cases of Collapsing Focal Segmental Glomerulosclerosis. Kidney Med. 2020 Jun 7;2(4):488-492. doi: 10.1016/j.xkme.2020.05.004. PMID: 32775989; PMCID: PMC7275990.

29: Corbett KS, Edwards DK, Leist SR, Abiona OM, Boyoglu-Barnum S, Gillespie RA, Himansu S, Schäfer A, Ziwawo CT, DiPiazza AT, Dinnon KH, Elbashir SM, Shaw CA, Woods A, Fritch EJ, Martinez DR, Bock KW, Minai M, Nagata BM, Hutchinson GB, Wu K, Henry C, Bahl K, Garcia-Dominguez D, Ma L, Renzi I, Kong WP, Schmidt SD, Wang L, Zhang Y, Phung E, Chang LA, Loomis RJ, Altaras NE, Narayanan E, Metkar M, Presnyak V, Liu C, Louder MK, Shi W, Leung K, Yang ES, West A, Gully KL, Stevens LJ, Wang N, Wrapp D, Doria-Rose NA, Stewart-Jones G, Bennett H, Alvarado GS, Nason MC, Ruckwardt TJ, McLellan JS, Denison MR, Chappell JD, Moore IN, Morabito KM, Mascola JR, Baric RS, Carfi A, Graham BS. SARS-CoV-2 mRNA vaccine design enabled by prototype pathogen preparedness. Nature. 2020 Oct;586(7830):567-571. doi: 10.1038/s41586-020-2622-0. Epub 2020 Aug 5. PMID: 32756549; PMCID: PMC7581537.

30: Shin D, Mukherjee R, Grewe D, Bojkova D, Baek K, Bhattacharya A, Schulz L, Widera M, Mehdipour AR, Tascher G, Geurink PP, Wilhelm A, van der Heden van Noort GJ, Ovaa H, Müller S, Knobeloch KP, Rajalingam K, Schulman BA, Cinatl J, Hummer G, Ciesek S, Dikic I. Papain-like protease regulates SARS-CoV-2 viral spread and innate immunity. Nature. 2020 Nov;587(7835):657-662. doi: 10.1038/s41586-020-2601-5. Epub 2020 Jul 29. PMID: 32726803; PMCID: PMC7116779.

31: Pfaender S, Mar KB, Michailidis E, Kratzel A, Boys IN, V'kovski P, Fan W, Kelly JN, Hirt D, Ebert N, Stalder H, Kleine-Weber H, Hoffmann M, Hoffmann HH, Saeed M, Dijkman R, Steinmann E, Wight-Carter M, McDougal MB, Hanners NW, Pöhlmann S, Gallagher T, Todt D, Zimmer G, Rice CM, Schoggins JW, Thiel V. LY6E impairs coronavirus fusion and confers immune control of viral disease. Nat Microbiol. 2020 Nov;5(11):1330-1339. doi: 10.1038/s41564-020-0769-y. Epub 2020 Jul 23. PMID: 32704094; PMCID: PMC7916999.

32: Zhu FC, Guan XH, Li YH, Huang JY, Jiang T, Hou LH, Li JX, Yang BF, Wang L, Wang WJ, Wu SP, Wang Z, Wu XH, Xu JJ, Zhang Z, Jia SY, Wang BS, Hu Y, Liu JJ, Zhang J, Qian XA, Li Q, Pan HX, Jiang HD, Deng P, Gou JB, Wang XW, Wang XH, Chen W. Immunogenicity and safety of a recombinant adenovirus type-5-vectored COVID-19 vaccine in healthy adults aged 18 years or older: a randomised, double-blind, placebo-controlled, phase 2 trial. Lancet. 2020 Aug 15;396(10249):479-488. doi: 10.1016/S0140-6736(20)31605-6. Epub 2020 Jul 20. PMID: 32702299; PMCID: PMC7836858.

33: Folegatti PM, Ewer KJ, Aley PK, Angus B, Becker S, Belij-Rammerstorfer S, Bellamy D, Bibi S, Bittaye M, Clutterbuck EA, Dold C, Faust SN, Finn A, Flaxman AL, Hallis B, Heath P, Jenkin D, Lazarus R, Makinson R, Minassian AM, Pollock KM, Ramasamy M, Robinson H, Snape M, Tarrant R, Voysey M, Green C, Douglas AD, Hill AVS, Lambe T, Gilbert SC, Pollard AJ; Oxford COVID Vaccine Trial Group. Safety and immunogenicity of the ChAdOx1 nCoV-19 vaccine against SARS-CoV-2: a preliminary report of a phase 1/2, single-blind, randomised controlled trial. Lancet. 2020 Aug 15;396(10249):467-478. doi: 10.1016/S0140-6736(20)31604-4. Epub 2020 Jul 20. Erratum in: Lancet. 2020 Aug 15;396(10249):466. Erratum in: Lancet. 2020 Dec 12;396(10266):1884. PMID: 32702298; PMCID: PMC7445431.

34: Codo AC, Davanzo GG, Monteiro LB, de Souza GF, Muraro SP, Virgilio-da-Silva JV, Prodonoff JS, Carregari VC, de Biagi Junior CAO, Crunfli F, Jimenez Restrepo JL, Vendramini PH, Reis-de-Oliveira G, Bispo Dos Santos K, Toledo-Teixeira DA, Parise PL, Martini MC, Marques RE, Carmo HR, Borin A, Coimbra LD, Boldrini VO, Brunetti NS, Vieira AS, Mansour E, Ulaf RG, Bernardes AF, Nunes TA, Ribeiro LC, Palma AC, Agrela MV, Moretti ML, Sposito AC, Pereira FB, Velloso LA, Vinolo MAR, Damasio A, Proença-Módena JL, Carvalho RF, Mori MA, Martins-de-Souza D, Nakaya HI, Farias AS, Moraes-Vieira PM. Elevated Glucose Levels Favor SARS-CoV-2 Infection and Monocyte Response through a HIF-1α/Glycolysis-Dependent Axis. Cell Metab. 2020 Sep 1;32(3):437-446.e5. doi: 10.1016/j.cmet.2020.07.007. Epub 2020 Jul 17. Erratum in: Cell Metab. 2020 Sep 1;32(3):498-499. PMID: 32697943; PMCID: PMC7367032.

35: Ayres JS. A metabolic handbook for the COVID-19 pandemic. Nat Metab. 2020 Jul;2(7):572-585. doi: 10.1038/s42255-020-0237-2. Epub 2020 Jun 30. PMID: 32694793; PMCID: PMC7325641.

36: Nishiga M, Wang DW, Han Y, Lewis DB, Wu JC. COVID-19 and cardiovascular disease: from basic mechanisms to clinical perspectives. Nat Rev Cardiol. 2020 Sep;17(9):543-558. doi: 10.1038/s41569-020-0413-9. Epub 2020 Jul 20. PMID: 32690910; PMCID: PMC7370876.

37: Apicella M, Campopiano MC, Mantuano M, Mazoni L, Coppelli A, Del Prato S. COVID-19 in people with diabetes: understanding the reasons for worse outcomes. Lancet Diabetes Endocrinol. 2020 Sep;8(9):782-792. doi: 10.1016/S2213-8587(20)30238-2. Epub 2020 Jul 17. Erratum in: Lancet Diabetes Endocrinol. 2020 Oct;8(10):e5. Erratum in: Lancet Diabetes Endocrinol. 2020 Nov;8(11):e6. PMID: 32687793; PMCID: PMC7367664.

38: Jackson LA, Anderson EJ, Rouphael NG, Roberts PC, Makhene M, Coler RN, McCullough MP, Chappell JD, Denison MR, Stevens LJ, Pruijssers AJ, McDermott A, Flach B, Doria-Rose NA, Corbett KS, Morabito KM, O'Dell S, Schmidt SD, Swanson PA 2nd, Padilla M, Mascola JR, Neuzil KM, Bennett H, Sun W, Peters E, Makowski M, Albert J, Cross K, Buchanan W, Pikaart-Tautges R, Ledgerwood JE, Graham BS, Beigel JH; mRNA-1273 Study Group. An mRNA Vaccine against SARS-CoV-2 - Preliminary Report. N Engl J Med. 2020 Nov 12;383(20):1920-1931. doi: 10.1056/NEJMoa2022483. Epub 2020 Jul 14. PMID: 32663912; PMCID: PMC7377258.

39: Gupta A, Madhavan MV, Sehgal K, Nair N, Mahajan S, Sehrawat TS, Bikdeli B, Ahluwalia N, Ausiello JC, Wan EY, Freedberg DE, Kirtane AJ, Parikh SA, Maurer

MS, Nordvig AS, Accili D, Bathon JM, Mohan S, Bauer KA, Leon MB, Krumholz HM, Uriel N, Mehra MR, Elkind MSV, Stone GW, Schwartz A, Ho DD, Bilezikian JP, Landry DW. Extrapulmonary manifestations of COVID-19. Nat Med. 2020 Jul;26(7):1017-1032. doi: 10.1038/s41591-020-0968-3. Epub 2020 Jul 10. PMID: 32651579.

40: Zhao X, Zheng S, Chen D, Zheng M, Li X, Li G, Lin H, Chang J, Zeng H, Guo JT. LY6E Restricts Entry of Human Coronaviruses, Including Currently Pandemic SARS-CoV-2. J Virol. 2020 Aug 31;94(18):e00562-20. doi: 10.1128/JVI.00562-20. PMID: 32641482; PMCID: PMC7459569.

41: Labò N, Ohnuki H, Tosato G. Vasculopathy and Coagulopathy Associated with SARS-CoV-2 Infection. Cells. 2020 Jun 30;9(7):1583. doi: 10.3390/cells9071583. PMID: 32629875; PMCID: PMC7408139.

42: Vabret N, Britton GJ, Gruber C, Hegde S, Kim J, Kuksin M, Levantovsky R, Malle L, Moreira A, Park MD, Pia L, Risson E, Saffern M, Salomé B, Esai Selvan M, Spindler MP, Tan J, van der Heide V, Gregory JK, Alexandropoulos K, Bhardwaj N, Brown BD, Greenbaum B, Gümüş ZH, Homann D, Horowitz A, Kamphorst AO, Curotto de Lafaille MA, Mehandru S, Merad M, Samstein RM; Sinai Immunology Review Project. Immunology of COVID-19: Current State of the Science. Immunity. 2020 Jun 16;52(6):910-941. doi: 10.1016/j.immuni.2020.05.002. Epub 2020 May 6. PMID: 32505227; PMCID: PMC7200337.

43: George PM, Wells AU, Jenkins RG. Pulmonary fibrosis and COVID-19: the potential role for antifibrotic therapy. Lancet Respir Med. 2020 Aug;8(8):807-815. doi: 10.1016/S2213-2600(20)30225-3. Epub 2020 May 15. PMID: 32422178; PMCID: PMC7228727.

44: Ronco C, Reis T, Husain-Syed F. Management of acute kidney injury in patients with COVID-19. Lancet Respir Med. 2020 Jul;8(7):738-742. doi: 10.1016/S2213-2600(20)30229-0. Epub 2020 May 14. PMID: 32416769; PMCID: PMC7255232.

45: Ziegler CGK, Allon SJ, Nyquist SK, Mbano IM, Miao VN, Tzouanas CN, Cao Y, Yousif AS, Bals J, Hauser BM, Feldman J, Muus C, Wadsworth MH 2nd, Kazer SW, Hughes TK, Doran B, Gatter GJ, Vukovic M, Taliaferro F, Mead BE, Guo Z, Wang JP, Gras D, Plaisant M, Ansari M, Angelidis I, Adler H, Sucre JMS, Taylor CJ, Lin B, Waghray A, Mitsialis V, Dwyer DF, Buchheit KM, Boyce JA, Barrett NA, Laidlaw TM, Carroll SL, Colonna L, Tkachev V, Peterson CW, Yu A, Zheng HB, Gideon HP, Winchell CG, Lin PL, Bingle CD, Snapper SB, Kropski JA, Theis FJ, Schiller HB, Zaragosi LE, Barbry P, Leslie A, Kiem HP, Flynn JL, Fortune SM, Berger B, Finberg RW, Kean LS, Garber M, Schmidt AG, Lingwood D, Shalek AK, Ordovas-Montanes J; HCA Lung Biological Network. Electronic address: lung-network@humancellatlas.org; HCA Lung Biological Network. SARS-CoV-2 Receptor ACE2 Is an Interferon-Stimulated Gene in Human Airway Epithelial Cells and Is Detected in Specific Cell Subsets across Tissues. Cell. 2020 May 28;181(5):1016-1035.e19. doi: 10.1016/j.cell.2020.04.035. Epub 2020 Apr 27. PMID: 32413319; PMCID: PMC7252096.

46: Berlin DA, Gulick RM, Martinez FJ. Severe Covid-19. N Engl J Med. 2020 Dec

17;383(25):2451-2460. doi: 10.1056/NEJMcp2009575. Epub 2020 May 15. PMID: 32412710.

47: Levi M, Thachil J, Iba T, Levy JH. Coagulation abnormalities and thrombosis in patients with COVID-19. Lancet Haematol. 2020 Jun;7(6):e438-e440. doi: 10.1016/S2352-3026(20)30145-9. Epub 2020 May 11. PMID: 32407672; PMCID: PMC7213964.

48: Zang R, Gomez Castro MF, McCune BT, Zeng Q, Rothlauf PW, Sonnek NM, Liu Z, Brulois KF, Wang X, Greenberg HB, Diamond MS, Ciorba MA, Whelan SPJ, Ding S. TMPRSS2 and TMPRSS4 promote SARS-CoV-2 infection of human small intestinal enterocytes. Sci Immunol. 2020 May 13;5(47):eabc3582. doi: 10.1126/sciimmunol.abc3582. PMID: 32404436; PMCID: PMC7285829.

49: Yu HQ, Sun BQ, Fang ZF, Zhao JC, Liu XY, Li YM, Sun XZ, Liang HF, Zhong B, Huang ZF, Zheng PY, Tian LF, Qu HQ, Liu DC, Wang EY, Xiao XJ, Li SY, Ye F, Guan L, Hu DS, Hakonarson H, Liu ZG, Zhong NS. Distinct features of SARS-CoV-2-specific IgA response in COVID-19 patients. Eur Respir J. 2020 Aug 27;56(2):2001526. doi: 10.1183/13993003.01526-2020. PMID: 32398307; PMCID: PMC7236821.

50: Hoffmann M, Kleine-Weber H, Pöhlmann S. A Multibasic Cleavage Site in the Spike Protein of SARS-CoV-2 Is Essential for Infection of Human Lung Cells. Mol Cell. 2020 May 21;78(4):779-784.e5. doi: 10.1016/j.molcel.2020.04.022. Epub 2020 May 1. PMID: 32362314; PMCID: PMC7194065.

51: Smieszek SP, Przychodzen BP, Polymeropoulos MH. Amantadine disrupts lysosomal gene expression: A hypothesis for COVID19 treatment. Int J Antimicrob Agents. 2020 Jun;55(6):106004. doi: 10.1016/j.ijantimicag.2020.106004. Epub 2020 Apr 30. PMID: 32361028; PMCID: PMC7191300.

52: Gordon DE, Jang GM, Bouhaddou M, Xu J, Obernier K, White KM, O'Meara MJ, Rezelj VV, Guo JZ, Swaney DL, Tummino TA, Hüttenhain R, Kaake RM, Richards AL, Tutuncuoglu B, Foussard H, Batra J, Haas K, Modak M, Kim M, Haas P, Polacco BJ, Braberg H, Fabius JM, Eckhardt M, Soucheray M, Bennett MJ, Cakir M, McGregor MJ, Li Q, Meyer B, Roesch F, Vallet T, Mac Kain A, Miorin L, Moreno E, Naing ZZC, Zhou Y, Peng S, Shi Y, Zhang Z, Shen W, Kirby IT, Melnyk JE, Chorba JS, Lou K, Dai SA, Barrio-Hernandez I, Memon D, Hernandez-Armenta C, Lyu J, Mathy CJP, Perica T, Pilla KB, Ganesan SJ, Saltzberg DJ, Rakesh R, Liu X, Rosenthal SB, Calviello L, Venkataramanan S, Liboy-Lugo J, Lin Y, Huang XP, Liu Y, Wankowicz SA, Bohn M, Safari M, Ugur FS, Koh C, Savar NS, Tran QD, Shengjuler D, Fletcher SJ, O'Neal MC, Cai Y, Chang JCJ, Broadhurst DJ, Klippsten S, Sharp PP, Wenzell NA, Kuzuoglu-Ozturk D, Wang HY, Trenker R, Young JM, Cavero DA, Hiatt J, Roth TL, Rathore U, Subramanian A, Noack J, Hubert M, Stroud RM, Frankel AD, Rosenberg OS, Verba KA, Agard DA, Ott M, Emerman M, Jura N, von Zastrow M, Verdin E, Ashworth A, Schwartz O, d'Enfert C, Mukherjee S, Jacobson M, Malik HS, Fujimori DG, Ideker T, Craik CS, Floor SN, Fraser JS, Gross JD, Sali A, Roth BL, Ruggero D, Taunton J, Kortemme T, Beltrao P, Vignuzzi M, García-Sastre A, Shokat KM, Shoichet BK, Krogan NJ. A SARS-CoV-2 protein interaction map reveals targets for drug repurposing. Nature. 2020 Jul;583(7816):459-468. doi: 10.1038/s41586-020-2286-9. Epub 2020 Apr 30. PMID: 32353859; PMCID: PMC7431030.

53: Long QX, Liu BZ, Deng HJ, Wu GC, Deng K, Chen YK, Liao P, Qiu JF, Lin Y, Cai XF, Wang DQ, Hu Y, Ren JH, Tang N, Xu YY, Yu LH, Mo Z, Gong F, Zhang XL, Tian WG, Hu L, Zhang XX, Xiang JL, Du HX, Liu HW, Lang CH, Luo XH, Wu SB, Cui XP, Zhou Z, Zhu MM, Wang J, Xue CJ, Li XF, Wang L, Li ZJ, Wang K, Niu CC, Yang QJ, Tang XJ, Zhang Y, Liu XM, Li JJ, Zhang DC, Zhang F, Liu P, Yuan J, Li Q, Hu JL, Chen J, Huang AL. Antibody responses to SARS-CoV-2 in patients with COVID-19. Nat Med. 2020 Jun;26(6):845-848. doi: 10.1038/s41591-020-0897-1. Epub 2020 Apr 29. PMID: 32350462.

54: Connors JM, Levy JH. COVID-19 and its implications for thrombosis and anticoagulation. Blood. 2020 Jun 4;135(23):2033-2040. doi: 10.1182/blood.2020006000. PMID: 32339221; PMCID: PMC7273827.

55: Verdecchia P, Cavallini C, Spanevello A, Angeli F. The pivotal link between ACE2 deficiency and SARS-CoV-2 infection. Eur J Intern Med. 2020 Jun;76:14-20. doi: 10.1016/j.ejim.2020.04.037. Epub 2020 Apr 20. PMID: 32336612; PMCID: PMC7167588.

56: Varga Z, Flammer AJ, Steiger P, Haberecker M, Andermatt R, Zinkernagel AS, Mehra MR, Schuepbach RA, Ruschitzka F, Moch H. Endothelial cell infection and endotheliitis in COVID-19. Lancet. 2020 May 2;395(10234):1417-1418. doi: 10.1016/S0140-6736(20)30937-5. Epub 2020 Apr 21. PMID: 32325026; PMCID: PMC7172722.

57: Hirano T, Murakami M. COVID-19: A New Virus, but a Familiar Receptor and Cytokine Release Syndrome. Immunity. 2020 May 19;52(5):731-733. doi: 10.1016/j.immuni.2020.04.003. Epub 2020 Apr 22. PMID: 32325025; PMCID: PMC7175868.

58: Ulrich H, Pillat MM. CD147 as a Target for COVID-19 Treatment: Suggested Effects of Azithromycin and Stem Cell Engagement. Stem Cell Rev Rep. 2020 Jun;16(3):434-440. doi: 10.1007/s12015-020-09976-7. PMID: 32307653; PMCID: PMC7167302.

59: Larsen CP, Bourne TD, Wilson JD, Saqqa O, Sharshir MA. Collapsing Glomerulopathy in a Patient With COVID-19. Kidney Int Rep. 2020 Apr 9;5(6):935-939. doi: 10.1016/j.ekir.2020.04.002. PMID: 32292867; PMCID: PMC7142700.

60: Malavolta M, Giacconi R, Brunetti D, Provinciali M, Maggi F. Exploring the Relevance of Senotherapeutics for the Current SARS-CoV-2 Emergency and Similar Future Global Health Threats. Cells. 2020 Apr 8;9(4):909. doi: 10.3390/cells9040909. PMID: 32276453; PMCID: PMC7226793.

61: Gheblawi M, Wang K, Viveiros A, Nguyen Q, Zhong JC, Turner AJ, Raizada MK, Grant MB, Oudit GY. Angiotensin-Converting Enzyme 2: SARS-CoV-2 Receptor and Regulator of the Renin-Angiotensin System: Celebrating the 20th Anniversary of the Discovery of ACE2. Circ Res. 2020 May 8;126(10):1456-1474. doi: 10.1161/CIRCRESAHA.120.317015. Epub 2020 Apr 8. PMID: 32264791; PMCID: PMC7188049.

62: Ou X, Liu Y, Lei X, Li P, Mi D, Ren L, Guo L, Guo R, Chen T, Hu J, Xiang Z, Mu Z, Chen X, Chen J, Hu K, Jin Q, Wang J, Qian Z. Characterization of spike glycoprotein of SARS-CoV-2 on virus entry and its immune cross-reactivity with SARS-CoV. Nat Commun. 2020 Mar 27;11(1):1620. doi: 10.1038/s41467-020-15562-9. Erratum in: Nat Commun. 2021 Apr 1;12(1):2144. PMID: 32221306; PMCID: PMC7100515.

63: Walls AC, Park YJ, Tortorici MA, Wall A, McGuire AT, Veesler D. Structure, Function, and Antigenicity of the SARS-CoV-2 Spike Glycoprotein. Cell. 2020 Apr 16;181(2):281-292.e6. doi: 10.1016/j.cell.2020.02.058. Epub 2020 Mar 9. Erratum in: Cell. 2020 Dec 10;183(6):1735. PMID: 32155444; PMCID: PMC7102599.

64: Hoffmann M, Kleine-Weber H, Schroeder S, Krüger N, Herrler T, Erichsen S, Schiergens TS, Herrler G, Wu NH, Nitsche A, Müller MA, Drosten C, Pöhlmann S. SARS-CoV-2 Cell Entry Depends on ACE2 and TMPRSS2 and Is Blocked by a Clinically Proven Protease Inhibitor. Cell. 2020 Apr 16;181(2):271-280.e8. doi: 10.1016/j.cell.2020.02.052. Epub 2020 Mar 5. PMID: 32142651; PMCID: PMC7102627.

65: Wrapp D, Wang N, Corbett KS, Goldsmith JA, Hsieh CL, Abiona O, Graham BS, McLellan JS. Cryo-EM structure of the 2019-nCoV spike in the prefusion conformation. Science. 2020 Mar 13;367(6483):1260-1263. doi: 10.1126/science.abb2507. Epub 2020 Feb 19. PMID: 32075877; PMCID: PMC7164637.

66: Léveillé M, Estall JL. Mitochondrial Dysfunction in the Transition from NASH to HCC. Metabolites. 2019 Oct 16;9(10):233. doi: 10.3390/metabo9100233. PMID: 31623280; PMCID: PMC6836234.

67: Kuwabara WMT, Yokota CNF, Curi R, Alba-Loureiro TC. Obesity and Type 2 Diabetes mellitus induce lipopolysaccharide tolerance in rat neutrophils. Sci Rep. 2018 Dec 3;8(1):17534. doi: 10.1038/s41598-018-35809-2. PMID: 30510205; PMCID: PMC6277411.

68: Ohishi M. Hypertension with diabetes mellitus: physiology and pathology. Hypertens Res. 2018 Jun;41(6):389-393. doi: 10.1038/s41440-018-0034-4. Epub 2018 Mar 19. PMID: 29556093.

69: Pardi N, Hogan MJ, Porter FW, Weissman D. mRNA vaccines - a new era in vaccinology. Nat Rev Drug Discov. 2018 Apr;17(4):261-279. doi: 10.1038/nrd.2017.243. Epub 2018 Jan 12. PMID: 29326426; PMCID: PMC5906799.

70: Thompson BT, Chambers RC, Liu KD. Acute Respiratory Distress Syndrome. N Engl J Med. 2017 Aug 10;377(6):562-572. doi: 10.1056/NEJMra1608077. PMID: 28792873.

71: Silberberg E, Filep JG, Ariel A. Weathering the Storm: Harnessing the Resolution of Inflammation to Limit COVID-19 Pathogenesis. Front Immunol. 2022 May 9;13:863449. doi: 10.3389/fimmu.2022.863449. PMID: 35615359; PMCID: PMC9124752.

72: Zumla A, Chan JF, Azhar EI, Hui DS, Yuen KY. Coronaviruses - drug discovery

and therapeutic options. Nat Rev Drug Discov. 2016 May;15(5):327-47. doi: 10.1038/nrd.2015.37. Epub 2016 Feb 12. PMID: 26868298; PMCID: PMC7097181.

73: Pandey S, Kawai T, Akira S. Microbial sensing by Toll-like receptors and intracellular nucleic acid sensors. Cold Spring Harb Perspect Biol. 2014 Oct 9;7(1):a016246. doi: 10.1101/cshperspect.a016246. PMID: 25301932; PMCID: PMC4292165.
